# Comparison of the protein-coding genomes of three deep-sea, sulfur-oxidising bacteria: “*Candidatus* Ruthia magnifica”, “*Candidatus* Vesicomyosocius okutanii” and *Thiomicrospira crunogena*

**DOI:** 10.1186/s13104-017-2598-5

**Published:** 2017-07-20

**Authors:** Susan E. McGill, Daniel Barker

**Affiliations:** 1 School of Biology, University of St Andrews, St Andrews, Fife KY16 9TH UK; 2 Institute of Evolutionary Biology, University of Edinburgh, Charlotte Auerbach Road, The Kings Buildings, Edinburgh, EH9 3FL UK

**Keywords:** “*Candidatus* Ruthia magnifica”, “*Candidatus* Vesicomyosocius okutanii”, *Thiomicrospira crunogena*, Thiotrichales, Sulfur-oxidising bacteria, Raspberry Pi, OrthoMCL, Comparative genomics, Paralogs, Orthologs

## Abstract

**Objective:**

**“** *Candidatus* Ruthia magnifica”, “*Candidatus* Vesicomyosocius okutanii” and *Thiomicrospira crunogena* are all sulfur-oxidising bacteria found in deep-sea vent environments. Recent research suggests that the two symbiotic organisms, “*Candidatus* R. magnifica” and “*Candidatus* V. okutanii”, may share common ancestry with the autonomously living species *T. crunogena*. We used comparative genomics to examine the genome-wide protein-coding content of all three species to explore their similarities. In particular, we used the OrthoMCL algorithm to sort proteins into groups of putative orthologs on the basis of sequence similarity.

**Results:**

The OrthoMCL inflation parameter was tuned using biological criteria. Using the tuned value, OrthoMCL delimited 1070 protein groups. 63.5% of these groups contained one protein from each species. Two groups contained duplicate protein copies from all three species. 123 groups were unique to *T. crunogena* and ten groups included multiple copies of *T. crunogena* proteins but only single copies from the other species. “*Candidatus* R. magnifica” had one unique group, and had multiple copies in one group where the other species had a single copy. There were no groups unique to “*Candidatus* V. okutanii”, and no groups in which there were multiple “*Candidatus* V. okutanii” proteins but only single proteins from the other species. Results align with previous suggestions that all three species share a common ancestor. However this is not definitive evidence to make taxonomic conclusions and the possibility of horizontal gene transfer was not investigated. Methodologically, the tuning of the OrthoMCL inflation parameter using biological criteria provides further methods to refine the OrthoMCL procedure.

## Introduction

“*Candidatus* Ruthia magnifica” strain Cm, “*Candidatus* Vesicomyosocius okutanii” HA and *Thiomicrospira crunogena* XCL-2 are all sulfur-oxidising bacteria found in deep-sea vent environments.

“*Candidatus* R. magnifica” and “*Candidatus* V. okutanii” live symbiotically in the gill epithelial cells of giant clam species: “*Candidatus* R. magnifica” in *Calyptogena magnifica* [1] and “*Candidatus* V. okutanii” in *Calyptogena okutanii* [2]. It is predicted that they are predominantly transmitted vertically via their host’s eggs [3, 4]. These hosts have reduced or vestigial digestive tracts and are therefore dependent on their symbionts for their nutritional requirements. As both giant clam species reside in deep-sea vent environments their symbionts are able to utilise the sulfur, produced by the vents, to provide their hosts with carbon and other nutrients [1, 2]. The symbionts’ dependence on the host varies, for example “*Candidatus* R. magnifica” encodes pathways to synthesise 20 amino acids [1], whereas “Candidatus V. okutanii” encodes pathways for 18 amino acids [2]. It has been hypothesised that missing essential genes in the symbiont may help maintain a stable symbiont population in a host cell [2].

Recent sequence-based reconstructions of phylogenetic trees suggest that “*Candidatus* R. magnifica” and “*Candidatus* V. okutanii” form a clade with each other, and a broader clade with *T. crunogena* [5, 6]*. T. crunogena* lives independently, though in the same deep-sea vent environments.

Preliminary to detailed studies on ancestry and adaptation among these three taxa, we can predict paralogs and orthologs across their genomes. Paralogs are genes arising by a duplication event within a species, and orthologs are genes in different taxa whose common ancestor is a gene present in the most recent common ancestral taxon [7, 8]. Although these definitions are explicitly phylogenetic, requiring a gene tree and a species tree, prediction of orthologous groups is often performed on the basis of sequence similarity alone. We investigated the evolution of the protein-coding gene content across all three species using OrthoMCL [9] and BLAST [10], to create protein groups based on sequence similarity, and UniProt [11], to assign functions to these groups.

Compared to an earlier comparative genomics study including the three species [12], our methodology allows more detailed investigation of variation in gene copy number. In contrast to purely reciprocal-best methods which predict only 1:1 orthologous relationships across taxa, OrthoMCL groups putative paralogs into orthologous groups of two or more sequences, imposing no upper limit on group size and no requirement that each group be present in each species.

## Main text

### Methods

The 4273π variant of the Raspbian Linux operating system [13] was used on a Raspberry Pi computer (Version 1, Model B, Revision 2.0). Genome-wide protein sets for “*Candidatus* Ruthia magnifica” strain Cm, “*Candidatus* Vesicomyosocius okutanii” HA and *Thiomicrospira crunogena* XCL-2 were downloaded, in FASTA format, from the Ensembl genomes database (http://ensemblgenomes.org) [14, 15]. OrthoMCL software (http://orthomcl.org) [9] and MCL [16] were used to delimit protein groups based on sequence similarity.

The OrthoMCL procedure was followed as outlined in the OrthoMCL user guide, with the exception of using the substitution matrix BLOSUM45 for the ‘all-versus-all’ NCBI BLAST [10] and omitting BLAST’s -z parameter; and for our final analysis, the inflation value (*I*) was set to 1.4 when running MCL.

As the inflation value decreases, sequences are included in fewer, larger groups, reducing the tightness and granularity of the delimited groups. To determine the optimal value of the inflation parameter, first a range of values were tested (*I* = 1.1, 1.2, 1.3, 1.4, 1.5, 1.6, 1.7, 1.8, 1.9, 2, 4, 6, 10 and 12). Annotated functions of the first (largest) three protein groups were examined using UniProt (http://uniprot.org) [11]. This revealed that only values 1.2–1.9 gave rise to groups that are both functionally cohesive and inclusive. Within this range, group 1, for example, contains diguanylate phosphodiesterases/cyclases (Table 1) but when *I* is increased to 2 these are split into different groups. Furthermore, when *I* = 1.1 proteins with different functions (transcriptional regulators—winged helix family) are also included within this group.

**Table 1:** Predicted protein functions of large or biologically interesting groups provided from OrthoMCL results

	Group no.	Total protein count	No. of proteins in “*Candidatus* R. magnifica”	No. of proteins in “*Candidatus* V. okutanii”	No. of proteins in *T. crunogena*	Proposed function
Groups unique to *T. crunogena*	1	40	0	0	40	Diguanylate phosphodiesterases/cyclases
	2	13	0	0	13	Transmembrane histidine kinases
	3	13	0	0	13	Methyl-accepting chemotaxis sensory transducers
	4	11	0	0	11	Two component response regulators
Groups with multiple copies in *T. crunogena* (but single in “*Candidatus* R. magnifica” and “*Candidatus* V. okutanii”)	5	8	1	1	6	Transcriptional regulators (Fis family)
	11	6	1	1	4	Ammonium transporters
	14	5	1	1	3	ABC transporters
	16	4	1	1	2	Bifunctional protein FolD
	17	4	1	1	2	Peptidyl-prolyl cis–trans isomerases
	18	4	1	1	2	Non-canonical purine NTP pyrophosphatases
	19	4	1	1	2	Peptidyl-prolyl cis–trans isomerases
	20	4	1	1	2	Cold-shock DNA-binding proteins
	21	4	1	1	2	Multicopper oxidases
	26	4	1	1	2	Lon proteases
Groups unique to *“Candidatus* R. magnifica”	746	2	2	0	0	Glycosyl transferases (family 2)
Groups with multiple copies in “*Candidatus* R. magnifica” (but single in “*Candidatus* V. okutanii” and *T.crunogena*)	27	4	2	1	1	Histidinol-phosphate aminotransferases
Groups with multiple copies in all three species	9	6	2	2	2	Elongation factors (Tu)
	10	6	2	2	2	Nitrogen regulatory proteins (P-II)

Group no. was assigned arbitrarily by OrthoMCL

Results using *I* = 1.2, 1.3 and 1.4 gave rise to a group which was not present using other values. It had multiple copies of “*Candidatus* R. magnifica” proteins but only single copies of “*Candidatus* V. okutanii” and *T. crunogena* proteins. UniProt suggested that these proteins had the same function (histidinol-phosphate aminotransferases) but increasing the *I* value split them up into different groups. Hence on biological grounds, *I* > 1.4 was rejected. *I* = 1.4 was used for the final analysis presented here, as it gave the strongest restraints on group formation while still maintaining this aminotransferase group (Table 1, Group 27).

Once groups were delimited, a Perl script, modified from [17], was used to count the number of times each species was represented in each protein group [15]. To verify the reliability of the script, our final OrthoMCL groups file was used as input for an independently-written protein-counting script (Kevin Kiesworo, unpublished) and the same counts were obtained. Additionally, the OrthoMCL groups file from a similar study on different taxa (Hannah Currant, unpublished) was used as input for our script, and the same counts were obtained as in that study.

Functions of both the largest and most interesting groups were then inferred by searching for protein accessions in UniProt: group members were searched until a common function was found between at least four proteins, or for smaller groups all members were searched (Table 1).

### Results

OrthoMCL predicted 1070 protein groups based on sequence similarity [15]. 63.5% of these contained a single protein from each of the three species. Two groups had duplicate protein copies in all three species (Table 1). *T. crunogena* had 123 unique groups, and multiple copies in ten groups that only had single copies in the other two species. “*Candidatus* R. magnifica” had one unique group, and had multiple copies in one group where the other species had a single copy. “*Candidatus* V. okutanii” had no unique groups. Nor did it have multiple copies in any groups that only had single protein copies from the other species. There were no groups that contained multiple protein copies from two species and one copy in the third (Table 2).

**Table 2:** Numbers of groups paralogous in one or two species

Species	No. of groups of two or more proteins unique to the given species	No. of groups with multiple copies in the given species but single copies in both other species	No. of groups with a single copy in the given species but multiple copies in both other species
*T. crunogena*	123	10	0
“*Candidatus* R. magnifica”	1	1	0
“*Candidatus* V. okutanii”	0	0	0

## Discussion

### Similarities between all three species

679 of the 1070 protein groups delimited by OrthoMCL (63.5%) contained a single protein copy in each of the three species. This high degree of similarity could be a consequence of common ancestry, horizontal transfer in a shared habitat, or most likely a mixture of both.

All three species inhabit deep-sea vent environments which are highly variable and have constantly fluctuating factors such as sulfur and carbon concentrations [18]. In order to survive, the organisms must possess methods that allow them to deal with such fluctuations. One shared process, for example, is their ability to oxidise the sulfur supplied by deep-sea vents to fix carbon for use in cellular functions.

Two groups were predicted to contain duplicate protein copies in all three species (Table 1, Groups 9 and 10). These are consistent with duplication in a common ancestor, with subsequent speciations, although our current work does not distinguish this from other possibilities such as horizontal transfer.

All three species have a duplicate copy of elongation factor Tu (Table 1, Group 9). These paralogs are found in all proteobacteria and it has been hypothesised, therefore, that this duplication event preceded the divergence of this phylum [19]. It has been shown that the *tuf* genes that encode these proteins undergo gene conversion [20] which inhibits any divergence, and therefore sub- or neo-functionalisation, of the two genes [21]. The persistence of the duplicate may therefore indicate high levels of expression. Detection of this known group is promising in regards to the reliability of our methods.

Each species also has a duplicate in the group of nitrogen regulatory proteins (P-II; Table 1, Group 10). One of these copies, in “*Candidatus* V. okutanii”, is the product of the *glnK* gene. This gene seems to be commonly duplicated in some sub-divisions of proteobacteria [22] and its evolution seems to be associated with that of the *amtB* ammonium transporter gene, to which it is physically and functionally linked [23]. Interestingly, these ammonium transporters make up Group 11 (Table 1) and although there are four copies in *T. crunogena,* the other species have no duplicates. This would be consistent with genome reduction due to a symbiotic lifestyle [2], although our current work cannot distinguish this with certainty.

### Unique to *T. crunogena*

A large number of protein groups (123) are found only in *T. crunogena* (Table 2). Also, in 10 groups, *T. crunogena* has multiple protein copies where the other species only have one copy each (Table 1). As the only independent-living species studied, *T. crunogena* may require a larger number of genes and proteins for survival. The other, symbiotic, organisms can rely on their hosts to provide some essential functions and, therefore, loss of some genes could prove to be energetically favourable [2]. There is also a lower total protein count for these species (976 and 937 protein sequences, compared to 2196 in *T. crunogena*).

### Unique to “*Candidatus* R. magnifica”

“*Candidatus* R. magnifica” has one unique group that consists of glycosyl transferases (Table 1, Group 746).

There was also one group delimited that had multiple “*Candidatus* R. magnifica” proteins and only single proteins from the other species (Table 1, Group 27). This is a group of histidinol-phosphate aminotransferases.

### Unique to “*Candidatus* V. okutanii”

“*Candidatus* V. okutanii” has no unique groups or paralogs.

## Conclusions

The number of unique protein groups found in *T. crunogena* may highlight its independent lifestyle that is very different from the other, symbiont, species. On the other hand, all three species shared many groups in common that could be indicative of a shared common ancestor, as was previously hypothesised [5, 6]. However, sequence-based orthology prediction is not sufficient to resolve taxonomy [24].

Methodologically, our work extends comparative genomics on the low-cost Raspberry Pi computer in two main ways. Firstly, three species were used, as opposed to two species in earlier studies [17, 25]. With faster, more recent versions of the hardware, such as the Raspberry Pi 3, even larger numbers of species would be possible.

Secondly, in our current study the OrthoMCL inflation parameter has been tuned using the biological criterion of functional coherence of the first (largest) three protein groups. This contrasts with algorithmic criteria used by, for example, [26] and [27], and may be generalizable to other methods for delimiting groups that also use MCL [16], for example Orthofinder [28]. There may be no universally optimal way to set the inflation parameter. However, biological criteria will always be valuable, whether used alone or to verify an algorithmic approach. Methodologically, combining biological criteria to guide the choice of inflation parameter with other refinements in family prediction (e.g. [29]) may be a promising future direction.

## Limitations

The method used only utilises sequence-based orthology prediction to produce protein groups, without phylogeny reconstruction, and so it is not sufficient to resolve taxonomy [24]. In accordance with the International Code of Nomenclature of Bacteria [30], other information such as metabolic and reproductive features must be known before formal taxonomy can be assigned.

We are also unable to rule out the possibility that the similarities in the protein coding content of these three genomes were due to horizontal gene transfer. It is thought that these events are less common as symbionts of vesicomyid clams (such as *Calyptogena magnifica* and *Calyptogena okutanii*) are found in their host oocytes—suggesting that vertical transmission is predominant [3, 4], presumably reducing opportunities for horizontal gene transfer. However, there is also evidence that lateral transmission, and therefore horizontal gene transfer events, can occur [31]. Analysis of horizontal transfer among these species and their relatives, including investigation of the detail of horizontal transfers [32], would be a promising future direction.

## Abbreviations

“*Candidatus* R. magnifica”: “*Candidatus* Ruthia magnifica”
“*Candidatus* V. okutanii”: “*Candidatus* Vesicomyosocius okutanii”
*T. crunogena*: *Thiomicrospira crunogena*
